# Is plant mitochondrial RNA editing a source of phylogenetic incongruence? An answer from *in silico* and *in vivo* data sets

**DOI:** 10.1186/1471-2105-9-S2-S14

**Published:** 2008-03-26

**Authors:** Ernesto Picardi, Carla Quagliariello

**Affiliations:** 1 Dipartimento di Biologia Cellulare, Università della Calabria, Arcavacata di Rende, (87036), Italy

## Abstract

**Background:**

In plant mitochondria, the post-transcriptional RNA editing process converts C to U at a number of specific sites of the mRNA sequence and usually restores phylogenetically conserved codons and the encoded amino acid residues. Sites undergoing RNA editing evolve at a higher rate than sites not modified by the process. As a result, editing sites strongly affect the evolution of plant mitochondrial genomes, representing an important source of sequence variability and potentially informative characters.

To date no clear and convincing evidence has established whether or not editing sites really affect the topology of reconstructed phylogenetic trees. For this reason, we investigated here the effect of RNA editing on the tree building process of twenty different plant mitochondrial gene sequences and by means of computer simulations.

**Results:**

Based on our simulation study we suggest that the editing ‘noise’ in tree topology inference is mainly manifested at the cDNA level. In particular, editing sites tend to confuse tree topologies when artificial genomic and cDNA sequences are generated shorter than 500 bp and with an editing percentage higher than 5.0%. Similar results have been also obtained with genuine plant mitochondrial genes. In this latter instance, indeed, the topology incongruence increases when the editing percentage goes up from about 3.0 to 14.0%. However, when the average gene length is higher than 1,000 bp (*rps3*, *matR* and *atp1*) no differences in the comparison between inferred genomic and cDNA topologies could be detected.

**Conclusions:**

Our findings by the here reported *in silico* and *in vivo* computer simulation system seem to strongly suggest that editing sites contribute in the generation of misleading phylogenetic trees if the analyzed mitochondrial gene sequence is highly edited (higher than 3.0%) and reduced in length (shorter than 500 bp).

In the current lack of direct experimental evidence the results presented here encourage, thus, the use of genomic mitochondrial rather than cDNA sequences for reconstructing phylogenetic events in land plants.

## Background

The term RNA editing was coined for the first time almost 20 years ago to describe the specific posttranscriptional transformation of the genetic message in the kinetoplast, the specialized mitochondrion of trypanosomes[1]. Since its initial discovery, RNA editing has been found to involve many apparently unrelated biochemically processes and to occur in a variety of eukaryotes, including animals, plants, some unicellular organisms and, in viruses as well (for a recent comprehensive review see[2] and references therein).

RNA editing specifically alters the nucleotide sequence of an RNA transcript, making it different from that of the DNA template. Therefore, the discovery of this process challenged the long-accepted dogma of a colinear genetic information flow.

On the basis of nucleotide by nucleotide sequence colinearity between the edited transcript and the DNA template, the RNA editing systems have been categorized into two major types [2].

In the ‘insertion/deletion’ editing type, nucleotide residues are added to and/or taken away from the gene-specified sequence. These insertions or deletions have been found mainly in mitochondria of the parasite *Trypanosoma*[1] and of the slime mold *Physarum *[3].

In the second RNA editing type, termed ‘substitution’ editing, the sequence of the edited transcript and its gene are colinear, but not identical. Different cases of simple base substitution such as the deamination reaction in which a cytosine (C) or an adenosine (A) is converted to an uridine (U) and an inosine (I) have been described in a wide range of species [2]. If such base changes occur in the coding region of mRNAs, the amino acid specificity, unpredictable from genomic codons, can be altered resulting in the synthesis of polypeptides more evolutionarily conserved and functionally competent.

In land plant mitochondria, mRNA editing is extensive in terms of both the range of transcripts affected and the density of editing per transcript. For instance, in mitochondria of *Arabidopsis thaliana* the *rps4* gene requires 15 C to U transitions at the mRNA level to express a functional S4 polypeptide, while 8 editing events occur in the *cox3* transcripts [4]. By contrast, in the *Magnolia* mitochondria 28 and 22 edits have been found in the *rps4* and *cox3* transcripts, respectively [5,6]. This restoration of evolutionarily conserved amino acids, as well as the creation of translation initiation codon by conversion of ACG into standard AUG start codons, has been interpreted as a strong support for the functional significance of the plant organellar RNA editing [7].

Mitochondrial genomes of land plants have an exceptionally low rate of substitutions compared with the counterpart of most other eukaryotes [8]. In reference to this scenario, editing sites might strongly affect the evolution of plant mtDNAs, representing an important source of sequence variability and potentially informative characters.

Previous comparative analyses on different mitochondrial genes across angiosperms revealed that, at genomic “editable sites”, C to T transitions are more frequent than any other potential substitution [9,10]. A similar evolutionary dynamic has also been confirmed for the *cox1* gene in gymnosperms [11]. Furthermore, these editing positions display at the mtDNA level characteristic nucleotide patterns composed almost exclusively of pyrimidines. Consequently, the editing sites of plant mitochondrial genes might represent a significant source of phylogenetic incongruence.

As emphasized by Hiesel et al. [12] cDNA rather than genomic DNA sequences of plant mitochondrial genes, should be preferred in phylogenetic analysis especially because the translation of the genomic DNA is not perfectly colinear with the corresponding sequence of the functional protein. Bowe and dePamphilis [13], according to their results on the evolution of plant mitochondrial *cox* genes affirmed, instead, that genomic sequences undergoing RNA editing are appropriate to be included in phylogenetic inferences, because the editing process operates at the transcriptional level and, thus, should not affect the historical information stored in the DNA sequences. Although the debate about the effect of RNA editing on phylogenetics is still ongoing as set forth earlier by Pesole et al. [9] and most recently by Szmidt et al. [11], no extensive study has been done up to now to test how much the RNA editing (C to U) actually affects the topology of the reconstructed phylogenetic trees.

In order to test the performance of the mitochondrial mRNA editing sites in phylogenetic inference, a controlled *in silico* environment in which the accuracy of tree reconstruction was checked on artificially generated multiple alignments has been set up.

Finally, the results of simulation data have been contrasted with those from twenty different multiple alignments of plant mitochondrial genes.

## Methods

### Simulation of plant mitochondrial genomic-like and cDNA-like sequences

The evolution of plant mitochondrial genomic-like sequences under increasing percentages of RNA editing was performed by the EdiPy program appropriately designed and written in Python programming language and executed on a Linux cluster (see Appendix A in Additional file 1) [14].

EdiPy program [15] takes as input a rooted phylogenetic tree with branch lengths expressed as mean number of substitutions per site, and a text file containing both the positions to be simulated in an editable fashion and the corresponding nucleotide equilibrium frequencies. The total number of editable sites per data set is calculated using a fixed percentage in the range from 1.0 to 10.0%.

Nucleotides subjected to RNA editing at the DNA level are supposed to follow the Tamura and Nei (TrN) model [16]. Remaining positions, defined as background sites, were in parallel simulated according to one of the following evolutionary models: Jukes-Cantor (JC) [17] or Hasegawa-Kishino-Yano (HKY) [18]. The high evolutionary rate for editing sites was set up according to previous results by Shields and Wolfe [10].

When HKY substitution models was selected, a transition/transversion rate ratio of 3 was assumed (corresponding to the mean value estimated by maximum likelihood from different plant mitochondrial genes analyzed in this work). In addition, the following equilibrium nucleotide frequencies were used: gA=gC=gG=gT=0.25 for the JC model, and gA=0.30, gC=0.20, gG=0.20, gT=0.30 for the HKY model.

EdiPy program was also employed to generate cDNA-like data sets by *in silico* transcription of the corresponding genomic-like sequences. During the transcription, EdiPy assumes that the C-to-T edit would work randomly taking into account the species-specificity and, thus, processed paralogs due to reverse transcription and reinsertion into the mitochondrial genome [14]. In our opinion, the assumption that all C's labelled as editable at genomic level would be replaced by U's in the mRNA might, in effect, be too restrictive or conservative and valid only for genes belonging to closely related plant species.

### Automated analysis of simulated data sets

Maximum likelihood (ML) trees were estimated from several mitochondrial genes belonging to various land plants (including data from Bowe and dePamphilis [13]) using PHYML program [19] under the general time reversible model (GTR) [20]. The corresponding average branch lengths were, then, employed to generate three different topologies of 12, 18 and 24 taxa using the stochastic speciation process described by Kuhner and Felsenstein [21]. Because this generating process makes trees molecular-clock-like, every branch length of each tree was multiplied by a gamma distributed factor, following the methodology of Guindon and Gascuel [22] (see Appendix B for details about topologies and the relative branch lengths in Additional file 1).

**Figure 1:**
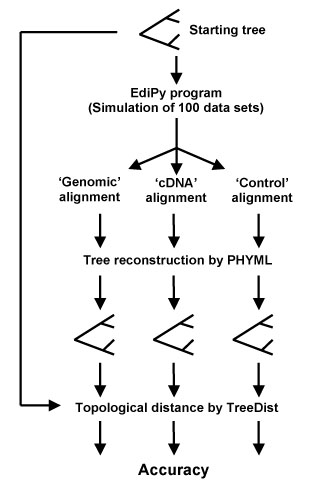
**Methodology overview.** Schematic overview of the methodology to *in silico* evaluate the effect of RNA editing on reconstructed tree topologies.

Each set of genomic and cDNA sequences was simulated 100 times and each replicate was submitted to PHYML program to estimate the ML phylogenetic tree under the HKY model of evolution [18,19]. Differences between inferred and ‘true’ trees were quantified by the topological distance using the Treedist program of the PHYLIP package [23]. The accuracy values of tree reconstruction were calculated for each data set as the proportion of correctly inferred topologies over the total number of detected trees. The general scheme of the methodology is shown in Figure 1.

### Retrieving and analyzing real plant mitochondrial genes

Sequences of plant mitochondrial genes and their corresponding cDNAs, with editing sites experimentally determined via direct cDNA sequencing, were downloaded from our specialized RNA editing database, REDIdb [24,25]. Each set of genomic and cDNA sequences was successively aligned using the ClustalW program with the default parameters and, manually adjusted, when necessary [26]. From a total of 42 collected plant mitochondrial protein-coding genes, any alignment that had less than 7 sequences was removed from the analyzed data sets. In this way, 20 alignments were kept for the purpose of the present work (more details are in the Additional file 1).

For each gene and cDNA alignment the editing percentage, the total number of variable sites and the corresponding evolutionary model, by AIC criterion [27,28], was obtained. The PHYML program was, then, used to reconstruct ML genomic and cDNA phylogenetic trees according to the detected models of nucleotide substitution [19].

The comparison between genomic and cDNA inferred trees was made by topological distance using the Treedist program [23]. This distance is currently defined as twice the number of interior branches at which sequence partition is different between the two trees compared. Yet, it is closely related to the number of internal branches and, thus, to the number of the analyzed sequences.

In order to compare results among all plant mitochondrial genes under study, a new simple measure was introduced. If *maxDt* was the maximum value of the topological distance and *Dt* was the truly detected topological distance, the *ratioDt* was then defined as:

$$ratioDt=\frac{Dt}{maxDt}$$

This ratio ranges from 0 to 1, depending on how much the inferred genomic and cDNA phylogenetic trees are topologically different. It approaches to 0 for identical trees and increases as the match worsens.

## Results

### Effect of editing at the genomic level

Given that the editing machinery acts directly on mRNAs, the information essential to direct the editing activity must be present at the mitochondrial genomic level [2]. For this reason, we investigated the effect of editing on phylogenetic inference at the genomic level, simulating around 200,000 sequences for approximately 140 million of nucleotides.

Five different sequence data sets of 200, 300, 500, 800 and 1,000 base pairs (bp) were generated along a tree of 12 taxa with percentages of editing sites ranging from 1.0 to 10.0% (see Appendix B for topology and relative branch lengths in Additional file 1). Background sites evolved under the JC model [17], whereas editing sites were simulated according to the TrN model of nucleotide substitution [16].

Table 1 summarize results of our *in silico* analyses (see ‘Genomic’ line), where the accuracy is shown as the probability of obtaining the correct tree.

**Table 1:** Accuracy of data sets generated along a 12 taxa tree (JC model). Accuracy of ML inferred trees from data sets generated along a 12 taxa tree and under a growing percentage of editing positions. Background sites evolved according to JC model [17]. Results from multiple alignments without editing sites are also shown as control.

*12 taxa tree - JC model*						
*% editing*	0	1	3	5	7	10
*200 nucleotides*								
*Genomic*	1	0.73	0.73	0.71	0.70	0.71*
*cDNA*	1	0.72	0.66	0.61	0.60	0.57
*Control*	1	0.73	0.72	0.69	0.63	0.65
*300 nucleotides*								
*Genomic*	1	0.91	0.91	0.89*	0.89*	0.88*
*cDNA*	1	0.9	0.85	0.78	0.76	0.71
*Control*	1	0.91	0.88	0.88	0.90	0.89
*500 nucleotides*								
*Genomic*	1	0.99	0.99	0.98	0.99*	0.96*
*cDNA*	1	0.99	0.97	0.94	0.91	0.86
*Control*	1	0.99	0.99	0.98	0.97	0.96
*800 nucleotides*								
*Genomic*	1	0.99	0.99	0.99	0.99	0.99
*cDNA*	1	0.99	0.99	0.97	0.96	0.95
*Control*	1	0.99	0.99	0.98	0.99	0.99
*1,000 nucleotides*								
*Genomic*	1	0.99	0.99	0.99	0.99	0.99
*cDNA*	1	0.99	0.99	0.99	0.99	0.97
*Control*	1	0.99	0.99	0.99	0.99	0.99

* indicates that the comparison between genomic and cDNA accuracy is significant (Pχ^2^_1_ < 0.05)

As expected, the presence of editing sites decreased the accuracy of reconstructed phylogenetic trees. In particular, the effect of editing sites was pronounced in the 200 and 300 bp data sets, even when only 1% of positions was subjected to RNA editing, that is, when only two nucleotides out of 200 were evolving in an editable fashion. However, in all cases examined the accuracy of the tree reconstruction was not less than 0.7, indicating that the presence of editing sites at the genomic level was not dramatically relevant at least for 7 out of 10 inferred trees.

When sequences were longer than 800 bp, the potential phylogenetic incongruence due to editing was hardly noticeable. Accuracy values close to 1 were, indeed, always found for alignments of 800 and 1,000 bp even when a high percentage of editing sites was simulated.

In a more realistic simulation, five additional data sets of 200, 300, 500, 800 and 1,000 bp were generated under the HKY evolutionary model [18]. As shown in Table 2 (see ‘Genomic’ line), the effect of fast evolving editing sites at genomic level did not significantly affect the tree reconstruction at least for sequences longer than 500 bp. Moreover, accuracy values were always higher than 0.80 even when genomic-like sequences were less than 500 bp and the editing percentage higher than 5.0%.

**Table 2:** Accuracy of data sets generated along a 12 taxa tree (HKY model). Accuracy of ML inferred trees from data sets generated along a 12 taxa tree and under a growing percentage of editing positions. Background sites evolved according to HKY model [18]. Results from multiple alignments without editing sites are also shown as control.

*12 taxa tree - HKY model*						
*% editing*	0	1	3	5	7	10
*200 nucleotides*								
*Genomic*	1	0.92	0.92	0.84	0.82*	0.80*
*cDNA*	1	0.89	0.85	0.80	0.74	0.72
*Control*	1	0.90	0.91	0.83	0.80	0.79
*300 nucleotides*								
*Genomic*	1	0.96	0.95	0.94	0.92*	0.90*
*cDNA*	1	0.93	0.93	0.91	0.83	0.77
*Control*	1	0.96	0.95	0.94	0.92	0.90
*500 nucleotides*								
*Genomic*	1	0.98	0.98	0.98	0.97	0.97
*cDNA*	1	0.98	0.98	0.96	0.95	0.92
*Control*	1	0.98	0.98	0.98	0.97	0.96
*800 nucleotides*								
*Genomic*	1	0.99	0.99	0.99	0.99	0.99
*cDNA*	1	0.99	0.99	0.99	0.98	0.97
*Control*	1	0.99	0.99	0.99	0.99	0.99
*1,000 nucleotides*								
*Genomic*	1	0.99	0.99	0.99	0.99	0.99
*cDNA*	1	0.99	0.99	0.99	0.98	0.98
*Control*	1	0.99	0.99	0.99	0.99	0.99

* indicates that the comparison between genomic and cDNA accuracy is significant (Pχ^2^_1_ < 0.05)

As clearly shown in Tables 1 and 2 (see ‘Genomic’ lines), the simulations performed under the more complex evolutionary model, HKY [18], rather than JC [17] gave the highest tree accuracy values. Moreover, the presence of editing sites at genomic level slightly affected the tree inference process when the number of simulated characters per data set decreased from 1,000 to 200 bp.

In Tables 3 and 4 (see ‘Genomic’ lines) are shown the results for the case of 1,000 bp data sets generated along 18 and 24 taxa trees under both JC and HKY evolutionary models (see Appendix B for topologies and relative branch lengths in Additional file 1). As previously observed in Tables 1 and 2 for alignments 800 and 1,000 bp long, the phylogenetic reconstruction was hardly affected even when the complexity of evolutionary models increased from JC to HKY (Tables 3, 4 and 5; Pχ^2^_1_< 0.05). Furthermore, similar results were observed when data sets longer than 1,000 bp were simulated along the 18 taxa tree and according to the simpler JC model [17] (Table 5; see ‘Genomic’ line).

**Table 3:** Accuracy of data sets generated along a 18 taxa tree (JC and HKY models). Accuracy of ML inferred trees from data sets of 1,000 bp generated along a 18 taxa tree and under a growing percentage of editing positions. Background sites evolved according both JC and HKY models [17,18]. Results from multiple alignments without editing sites are also shown as control.

*18 taxa tree - JC model*						
*% editing*	0	1	3	5	7	10
*1,000 nucleotides*								
*Genomic*	1	0.91	0.90*	0.93*	0.92*	0.88*
*cDNA*	1	0.86	0.75	0.65	0.52	0.46
*Control*	1	0.91	0.88	0.94	0.89	0.88
*18 taxa tree – HKY model*						
*% editing*	0	1	3	5	7	10
*1,000 nucleotides*								
*Genomic*	1	0.93	0.96*	0.96*	0.94*	0.93*
*cDNA*	1	0.87	0.86	0.85	0.73	0.71
*Control*	1	0.91	0.90	0.95	0.83	0.83

* indicates that the comparison between genomic and cDNA accuracy is significant (Pχ^2^_1_< 0.05)

**Table 4:** Accuracy of data sets generated along a 24 taxa tree (JC and HKY models). Accuracy of ML inferred trees from data sets of 1,000 bp generated along a 24 taxa tree and under a growing percentage of editing positions. Background sites evolved according both JC and HKY models [17,18]. Results from multiple alignments without editing sites are also shown as control.

*24 taxa tree - JC model*						
*% editing*	0	1	3	5	7	10
*1,000 nucleotides*								
*Genomic*	1	0.92	0.86	0.88*	0.80*	0.79*
*cDNA*	1	0.88	0.82	0.77	0.64	0.58
*Control*	1	0.95	0.88	0.91	0.90	0.86
*24 taxa tree – HKY model*						
*% editing*	0	1	3	5	7	10
*1,000 nucleotides*								
*Genomic*	1	0.94	0.94	0.91*	0.90*	0.90*
*cDNA*	1	0.93	0.92	0.83	0.78	0.73
*Control*	1	0.94	0.90	0.87	0.81	0.70

* indicates that the comparison between genomic and cDNA accuracy is significant (Pχ^2^_1_< 0.05)

**Table 5:** Accuracy of data sets generated along a 18 taxa tree (JC model). Accuracy of ML inferred trees from data sets of 1,500 bp generated along a 18 taxa tree and under a growing percentage of editing positions. Background sites evolved according to the JC model [17]. Results from multiple alignments lacking editing sites are also shown as control.

*18 taxa tree - JC model*						
*% editing*	0	1	3	5	7	10
*1,500 nucleotides*								
*Genomic*	1	0.98*	0.95*	0.95*	0.95*	0.93*
*cDNA*	1	0.89	0.81	0.77	0.65	0.56
*Control*	1	0.97	0.95	0.97	0.96	0.91

* indicates that the comparison between genomic and cDNA accuracy is significant (Pχ^2^_1_< 0.05)

### Differences between genomic and cDNA sequences

As shown in Table 1 (see ‘Genomic’ and ‘cDNA’ lines) for the case of alignments generated along the 12 taxa tree and under the JC model [17], accuracy values from genomic and cDNA inferred trees were roughly the same for sequences longer than 800 bp. On the contrary, the effect of RNA editing on tree reconstruction became relevant when the percentage of editing sites was higher than 5.0% and alignments were shorter than 500 bp (Table 1). Unlike results from artificial genomic data sets, the accuracy of trees deduced by cDNA sequences could also assume values below 0.6 (Table 1).

When artificial alignments were generated according to the HKY evolutionary models [18] slight differences between genomic and cDNA accuracy values could be recovered, especially for data sets longer than 500 bp (Table 2; see ‘Genomic’ and ‘cDNA’ lines). However, significant accuracy reduction for the cDNA inferred trees was found in data sets of 200 and 300 bp, but only for editing percentages higher than 5.0% (Table 2).

Interestingly, as reported in Tables 3 and 4 (see ‘Genomic’ and ‘cDNA’ lines), a major effect of RNA editing on phylogenetic inference process was established when cDNA sequences were generated along trees of 18 and 24 taxa and according to both the JC and HKY models. In particular, relevant effects emerged when cDNA sequences were simulated under editing percentages higher than 3.0%.

Moreover, as reported in Table 5, the extension of sequence length to 1,500 bp led to a very low reliability of cDNA inferred trees. In all cases examined differences in accuracy values between genomic and cDNA deduced topologies were significant (Pχ^2^_1_< 0.05).

As a control of both editing effect on tree inference and simulation analysis, additional data sets were generated excluding editing sites. In these cases shown in Tables 1,2, 3, 4 and 5 (see ‘Control’, ‘Genomic’ and ‘cDNA’ lines), accuracy values of tree topologies estimated from data sets without editing sites were closely related to those obtained from trees deduced by genomic-like sequences.

### RNA editing on real plant mitochondrial genes

Genomic and cDNA sequences of 42 different plant mitochondrial genes have been retrieved from our specialised RNA editing database, REDIdb [24]. Since in many cases, the number of available sequences was very small, we excluded from our study any plant mitochondrial gene with less than 7 sequences in the corresponding multiple alignment. Only a total of 20 genes (Table 6) were found to conform to this condition and were retained for the analysis. As shown in Table 6, 5 mitochondrial genes are longer than 1,000 bp (*atp1*, *matR*, *rps3*, *nad5*, *cob*), whereas 4 genes are shorter than 500 bp (*rps12*, *rps13*, *nad3*, *atp9*). The most edited gene is the *nad3* with 14% of its coding region altered by C to T post-transcriptional conversions. In contrast, the mitochondrial *atp1* gene is the least edited, with only 0.85% alterations.

**Table 6:** Plant mitochondrial genes used in this study and *ratioDt* values. For each gene, the number of sequences (N), the mean length (L), the editing percentage (E), the number of variable sites (Vg for genomic and Vc for cDNA), the evolutionary model (Mg for genomic and Mc for cDNA) and the *ratioDt* are shown.

Gene	N	L	E	Vg	Vc	Mg	Mc	*ratioDt*
*atp1*	7	1527.86	0.85	199	202	HKY+I	HKY+I	0
*matR*	8	2023.50	1.24	360	356	K80+G	K80+G	0
*atp8*	8	504.00	2.58	143	142	HKY+G	HKY+G	0
*rps3*	7	1670.14	2.93	483	472	GTR+I	GTR+I	0
*nad1*	7	980.57	4.90	71	63	HKY+I	HKY	0
*ccb3*	7	735.43	6.66	116	107	HKY+I	HKY+I	0
*atp6*	9	962.00	3.43	510	485	HKY+G+I	HKY+G+I	0.16
*cox3*	9	802.67	3.86	71	70	GTR+I	GTR+G	0.16
*rps12*	14	377.79	4.24	79	74	HKY+G	HKY+G	0.18
*nad5*	7	2012.14	1.99	139	124	HKY+I	HKY+I	0.25
*rpl5*	8	568.13	2.46	186	182	GTR+I	HKY+G	0.25
*atp4*	7	602.57	3.65	153	157	HKY+G	HKY+G	0.25
*nad6*	7	642.43	4.05	124	119	F81+I	F81+I	0.25
*rps13*	8	350.25	2.86	45	45	F81+G	F81+G	0.4
*cob*	10	1183.50	3.72	135	129	HKY+I	HKY+I	0.43
*cox2*	16	774.75	4.26	176	160	HKY+G	HKY+G	0.5
*ccb2*	7	621.00	11.27	75	72	HKY	HKY+G	0.5
*nad3*	19	363.00	14.05	86	72	GTR+G	GTR+G	0.5
*atp9*	16	234.19	6.41	81	72	K80+G	HKY+G	0.54
*nad9*	9	606.00	2.81	58	52	HKY+I	HKY+G	0.6

*Notes*. K80, Kimura; F81, Felsenstein 1981; HKY, Hasegawa-Kishino-Yano; GTR, General time reversible; I, invariant sites; G, gamma correction.

The editing percentage is calculated for each multiple alignment as the proportion of site patterns containing at least one editing event over the total number of site patterns.

Furthermore, for each genomic and cDNA multiple alignment the best model of nucleotide substitution has been detected by the AIC criterion [27,28]. In 14 out of 20 cases shown in Table 6, genomic DNA sequences followed the same evolutionary model identified for cDNA sequences.

**Figure 2:**
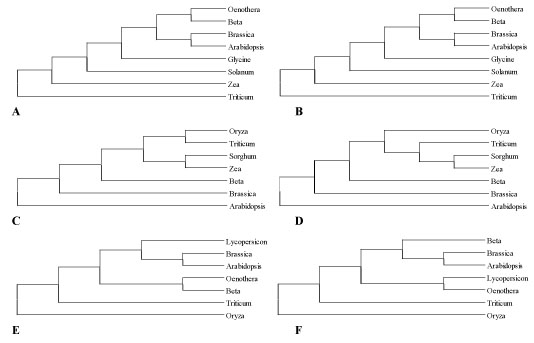
**Examples of genomic and cDNA trees from plant mitochondrial genes.** Genomic and cDNA tree topologies for three plant mitochondrial genes with different *ratioDt* values. A, B) Genomic and cDNA inferred trees for the *atp1* gene where *ratioDt* is equal to 0; C, D) genomic and cDNA topologies deduced for the *atp4* gene where there is a moderate editing bias (*ratioDt* equal to 0.25); E, F) genomic and cDNA trees for the *ccb2* gene where there is a strong editing effect (*ratioDt* equal to 0.50).

Genomic and cDNA inferred trees have been compared by topological distance. In addition, since our multiple alignments have a variable number of sequences per gene and, thus, a different maximum value of topological distance, we chose to define the *ratioDt* as main measure to compare DNA and cDNA deduced trees. This ratio is easily calculated from the observed topological distance divided by the maximum value that it could assume. In this way, a *ratioDt* of 0 is expected for completely identical inferred genomic and cDNA topologies, whereas a *ratioDt* equal to 1 is expected for trees in which the match is radically lost (Figure 2).

According to the *ratioDt*, the maximum number of differences between deduced genomic and cDNA trees was observed for *nad9* gene.

In general, as shown in Table 6, the *ratioDt* increased when the editing percentage went up from about 3.0 to 14.0%. In particular, when the average gene length was higher than 1,000 bp (*rps3*, *matR* and *atp1*) no differences could be detected in the comparison between inferred genomic and cDNA topologies. On the contrary, genes shorter than 1,000 bp showed heterogeneous *ratioDt* values depending on the editing percentages and the total number of variable characters.

Surprisingly, we detected *ratioDt* values greater than 0 for the mitochondrial genes *nad5* and *cob*, in which the mean sequence length was higher than 1,000 bp and the editing percentage was 1.9 and 3.7%, respectively.

Finally, while comparing the number of variable sites for each gene and the corresponding cDNA, it became evident that in general genomic sequences generate a higher phylogenetic signal than their transcripts. Nonetheless, in none of the investigated cases the increased genomic phylogenetic signal was statistically significant (Pχ^2^_1_> 0.05).

## Discussion

### Phylogenetic incongruence and editing simulation

RNA editing in plant mitochondria remodels mitochondrial precursor mRNAs via specific C to U conversions [2,29]. As a consequence, the genetic information in the transcript may differ from that one of the gene [29] leading to conflicting phylogenetic tree topologies. Although there are several reasons to account for the phylogenetic incongruence caused by the RNA editing process, three of them should at least be invoked.

The first and foremost among these is that plant mitochondrial sites subjected to RNA editing might negatively alter and affect sequence nucleotide stationarity because they are exclusively constituted of pyrimidines [30]. This might be especially true when editing sites within the same gene are heterogeneously distributed across different land plants or in instances of massive RNA editing reduction. As an explanatory example, the mitochondrial *cox3* gene in *Magnolia* requires 22 edits to yield a functional protein product, whereas only 13 and 10 editing events have been observed in the same gene of wheat and olive tree mitochondria, respectively [5,31].

Second, editing sites tend to have a more accelerated evolutionary rate than sites not subjected to RNA editing as demonstrated by the comparison of the substitution number per site in different land plant mitochondrial sequences [10]. Therefore, mitochondrial genomic and cDNA sequences exhibit different evolutionary dynamics. [9]. Third, over great evolutionary distances, editing sites might conceivably become saturated for multiple changes, given the rapid turnover of pyrimidines [10].

Nonetheless, the edited plant mitochondrial sequences are currently used in phylogenetics to provide insights into relationships at all levels in the green plant hierarchy of life [32-35]. The question remains, thus, as to how much the presence of editing sites will affect phylogenetic analysis of mitochondrial sequences and which sequences should be used, genomic or cDNA?

According to Hiesel et al. [12] cDNA sequences should be used in phylogenetics of land plants, because they are sequenced from mRNA and predict the true protein sequence. Unfortunately, Hiesel et al. [12] did not show trees deduced from genomic DNA sequences, omitting a discussion about the effect of editing on phylogenetic analysis.

By contrast, Bowe and dePamphilis [13] as well as Szmidt et al. [11], argued that genomic DNA sequences are at least as useful as cDNA sequences for reconstructing phylogenetic events. The editing effect was qualitatively evaluated comparing deduced mitochondrial genomic and cDNA trees [11,13].

A major question to be considered is the potentially misleading choice of which data set, genomic or cDNA, has to be used in phylogenetic analysis if plant mitochondrial genomic and cDNA trees are not similar. It became difficult, indeed, to judge which tree, genomic or cDNA, is closer to the correct one when it is not possible to establish with confidence the true phylogenetic relationships between homologous sequences.

Currently numerical simulations are employed for studying the accuracy of different molecular biological processes under idealized conditions, which are especially useful to exhaustively explore the effect of multiple parameters affecting the performance of methods of phylogenetic inference [36]. In particular, the simulation of plant mitochondrial genes subjected to RNA editing allows us to establish either the editing effect on tree reconstruction is more extensive at the genomic than the cDNA level or the number of topological differences between deduced genomic and cDNA trees.

The basic assumption of our simulation is that sites subjected to RNA editing evolve differently than the remaining sites, defined as background, evolving specifically according to the TrN nucleotide substitution model [16] (see the Methods section).

Following our experimental design, the ‘noise’ introduced by a growing percentage of editing sites should be evident in terms of reduction of topological accuracy. The variability range for the percentage values of editing sites was fixed from 1.0 to 10.0% because these values are roughly the same detected for real plant mitochondrial genes (see Table 6). Moreover, percentage values higher than 10.0% might not mimic real plant mitochondrial editing patterns and, thus, substantially alter the nucleotide composition per sequence.

Our results show that when background sites are generated under the simpler JC evolutionary model [17], the editing bias at genomic level is more evident for short and highly edited sequence data sets (Table 1). Nonetheless, a slight reduction of tree accuracy is also manifest when background sites are simulated according to a more complex substitution model as the HKY [18] (Table 2). This finding is not surprising given that the tree reconstruction is performed under the ML criterion using the more complicated HKY model that takes into account variable nucleotide frequencies and different rates for transitions and transversions. In all cases, including also simulations along the 18 and 24 taxa trees, the accuracy of tree reconstruction at genomic level is comparable with that one obtained in previous simulation studies performed to evaluate the ability of different methods in phylogenetic tree inference [37,38] (Tables 3, 4 and 5). However, in function of the number of simulated editing sites, a minimal decrease of efficiency in tree reconstruction is often found, indicating that the editing ‘noise’ is generally present and associated with high editing percentages, even though it is not so relevant at genomic level. It is likely that editing ‘noise’ is partially due to the reduction of the character-state space at level of editing sites. It has been clearly demonstrated that a relatively little increase in character-state space can provide enormous benefits for the accuracy of phylogenetic inference [39].

On the other hand, simulation results for data sets lacking editing sites clearly indicate that the elimination of edits lead to tree topologies close to those inferred by genomic-like data sets (Tables 1, 2, 3, 4 and 5).

However, it should be noted that our simulations greatly rely on the model tree topology used to generate the sequence data. Since only few randomly generated topologies have been used, our observations may be limited to topologies of the same type. Furthermore, the complexity of the actual nucleotide substitution pattern poses the problem of the model choice. Even sophisticated models tend to oversimplify the real evolutionary patterns. Therefore, given the limiting nature of the numerical simulations, our results represent only the simplest expectation of the RNA editing effect on tree reconstruction.

### Comparison between artificial and real data sets

In contrast with previously published studies by Bowe and dePamphilis [13], Pesole et al. [9] and Szmidt et al. [11], in which only a limited number of characters and taxa was investigated to verify the effect of editing on phylogenetic reconstruction, here 20 different plant mitochondrial genes have been analyzed.

As shown in Table 6, when the model describing the process of nucleotide substitutions for genomic and cDNA sequences is valuated separately, in 14 out of 20 genes, genomic and cDNA sequences followed the same evolutionary model, suggesting that most likely the number of editing events per gene is not adequate to improve significantly the likelihood scores and, thus, to affect the choice of the best-fit evolutionary model [28].

Differently to the previous works of Bowe and dePamphilis [13] and Szmidt et al. [11], the phylogenetic bias due to RNA editing sites has been quantitatively valuated comparing ML genomic and cDNA inferred trees by means of the *ratioDt*. In a large number of plant mitochondrial genes analysed here the *ratioDt* assumes higher values when the percentage of editing sites increases (Table 6). If we consider that genomic and cDNA sequences diverge only by editing sites, conflicting tree topologies are most likely due exclusively to the presence of RNA editing sites (Figure 2). On the other hand, bearing in our mind that the aim of our work was to quantify the conflict between genomic and cDNA inferred topologies, any discussion about the systematic correctness of each deduced tree has been here deliberately omitted. Moreover, it should be mentioned that our inferred trees, technically called gene-trees, represents only the evolutionary relationships among genomic or cDNA sequences of each specific gene that, thus, might not be completely comparable with specie-trees (Figure 2).

As predicted by our *in silico* analyses (Tables 1, 2, 3, 4 and 5), when mitochondrial gene sequences are shorter than or around 500 bp the *ratioDt* values range from 0.18 to 0.54, indicating from moderate to serious corruption of tree reconstruction. In contrast, when analyzed gene sequences are longer than 800 bp (*atp1*, *matR*, *rps3* and *atp6*) the *ratioDt* is close to 0, indicating a perfect accord between genomic and cDNA inferred trees. Only in few examples where the number of sequences per gene is more than 14, as *cox2*, *nad3* and *atp9*, the *ratioDt* assumes the highest values ranging from 0.5 to 0.54 (Figure 2).

Although computer simulation model let us predict a potential effect of editing on the topology of many plant mitochondrial genes, misleading predictions might still be experienced. Indeed, artificial sequences are generated under simplified conditions and even when more complex models of evolution are invoked it is difficult to perfectly describe the real biological world. Other factors such as site rate variation and interdependence among sites should be taken into account [40,41]. For example, 5′- sequences adjacent to the editing sites might be required for RNA editing [42].

Misleading results could also emerge during the process of tree reconstruction, because the efficiency of ML methods of tree building depends also on the number of characters and taxa analyzed and on the number of variable sites [38]. In effect, when total site variability was not sufficient to reconstruct phylogenies, as for *nad5*, *cob* and *nad9* genes, the behaviour of editing sites might not be easily predictable by simulation.

Above all Bowe and dePamphilis emphasized [13] that processed paralogs, i.e. sequences due to reverse transcription and reinsertion into either the mitochondrial or nuclear genome as edited cDNA, critically affect the tree building process.

If processed paralogs become inserted into the mitochondrial genome, they certainly generate variability in the total number of editing sites per gene, a phenomenon also well known as species-specificity of RNA editing. In the latter event, the phylogenetic editing ‘noise’ can be straightforwardly evaluated by either the *in silico* or the *in vitro* approach according to the methodology described in the Methods section of this paper.

If processed paralogs become instead inserted into the nuclear genome they evolve in accordance with nuclear sequences, that is much faster than plant chloroplast and mitochondrial sequences [43]. Either way paralogs may really cause a critical phylogenetic incongruence [13].

## Conclusions

Studying the correlated rates of synonymous site evolution across plant genomes, Eyre-Walker and Gaut [44] wrote “RNA editing is a potential complication in the analysis of plant mitochondrial and chloroplast genes…. caution must be taken to ensure that … all edited sites are excluded from an analysis.”

In light of this statement and agreement with our results from simulated and genuine mitochondrial data sets, we conclude that:

• The editing ‘noise’ in the tree inference is mainly manifested at the cDNA level.

• Editing sites can contribute in generating misleading phylogenetic trees if the analyzed mitochondrial gene sequence is highly edited (higher than 3.0%) and reduced in length (shorter than 500 bp).

Although the removal of editing sites can contribute to reduce confusing the tree inferences when the plant mitochondrial genomic and cDNA sequences are combined [13], to the best of our knowledge, there is no evidence up to now that mitochondrial DNA sequences are misleading in phylogenetic analyses. Therefore, our findings favour the conclusion that mitochondrial genomic rather than cDNA sequences should be used for reconstructing phylogenetic events in land plants.

## Competing interests

The authors declare that they have no competing interests

## Authors' contributions

EP and CQ conceived this study. EP wrote the Python script used in this study, performed all the analyses and drafted the manuscript (text and figures).

CQ contributed substantially to the final manuscript. Both authors read and approved the final manuscript.

## Supplementary Material

Additional file 1 Portable Document Format
Additional_file1.pdf contains info about genes used in this study and two Appendices (A and B) with supplementary details related to EdiPy program and tree topologies used in simulations. Click here for file
