# Supplementary material for: Is plant mitochondrial RNA editing a source of phylogenetic incongruence? An answer from in silico and in vivo data sets
Source: BMC Bioinformatics. 2008 Mar 26;9(Suppl 2):S14. doi: 10.1186/1471-2105-9-S2-S14 (PMC2323663; doi:10.1186/1471-2105-9-S2-S14)
Supplement: Additional file 1 — Portable Document Format Additional_file1.pdf contains info about genes used in this study and two Appendices (A and B) with supplementary details related to EdiPy program and tree topologies used in simulations. [file 1471-2105-9-S2-S14-S1.pdf]

List of plant mitochondrial genes used in this study:

Genes	Sequences	Mean length	Editing percentage
atp1	7	1527.86	0.85
matR	8	2023.50	1.24
atp8	8	504.00	2.58
rps3	7	1670.14	2.93
nad1	7	980.57	4.90
ccb3	7	735.43	6.66
atp6	9	962.00	3.43
cox3	9	802.67	3.86
rps12	14	377.79	4.24
nad5	7	2012.14	1.99
rpl5	8	568.13	2.46
atp4	7	602.57	3.65
nad6	7	642.43	4.05
rps13	8	350.25	2.86
cob	10	1183.50	3.72
cox2	16	774.75	4.26
ccb2	7	621.00	11.27
nad3	19	363.00	14.05
atp9	16	234.19	6.41
nad9	9	606.00	2.81

The editing percentage is calculated for each multiple alignment as the proportion of site patterns containing at least one editing event over the total number of site patterns.

Appendix A

EdiPy is available by authors upon request or, alternatively, it can be used on-line at the following web page: http://biologia.unical.it/py_script/index.html. EdiPy can run on whatever platform provided of Python interpreter (at least version 2.3) and Numeric module [14].

Appendix B

Trees and relative branch lengths used for simulations are provided in Newick format as follow:

12 taxa tree

((((T12:0.050342,T8:0.0301736):0.013584,T1:0.011636):0.0348676,((T3:0.005542,T6:0.011
8624):0.0372248,T2:0.014572):0.0365044):0.0329892,((T4:0.0182456,T5:0.0574204):0.0240
032,(T11:0.0357984,((T10:0.0116456,T9:0.0399844):0.0245788,T7:0.020912):0.0187456):0.
0336708):0.0025172);

18 taxa tree

((((T17:0.0515495,T18:0.035304):0.0162625,(T3:0.0140595,T4:0.008812):0.0145515):0.032
3555,((T11:0.029959,T12:0.0438415):0.008869,T1:0.0270665):0.012289):0.0091555,((((T9:
0.0513715,T10:0.0333935):0.0249595,(T15:0.0138605,T16:0.0377015):0.0151685):0.02214
15,(T7:0.0396135,T8:0.016999):0.020538):0.022816,(((T5:0.0025265,T6:0.002508):0.02412
8,(T13:0.0065525,T14:0.006464):0.0312225):0.0422945,T2:0.0137415):0.028193):0.050989
9);

24 taxa tree

(((T1:0.133795,(T23:0.001892,T24:0.002000):0.135488):0.024121,(((T13:0.018254,T14:0.01
8429):0.041514,T5:0.059898):0.037671,(T15:0.014466,T16:0.014097):0.082975):0.061977):
0.039464,((((T6:0.048751,((T9:0.030342,T10:0.029709):0.010513,T8:0.040896):0.006757):0
.041584,(T11:0.021047,T12:0.020510):0.067881):0.012670,(T2:0.064097,T3:0.063507):0.03
5400):0.048746,(((T17:0.012142,T18:0.012170):0.032758,T7:0.045084):0.049591,((T4:0.06
0269,(T19:0.006039,T20:0.006564):0.053260):0.009095,(T21:0.004220,T22:0.004298):0.06
5208):0.023291):0.056861):0.045604);
